# Effect of Inulin Source and a Probiotic Supplement in Pig Diets on Carcass Traits, Meat Quality and Fatty Acid Composition in Finishing Pigs

**DOI:** 10.3390/ani11082438

**Published:** 2021-08-19

**Authors:** Eugeniusz R. Grela, Małgorzata Świątkiewicz, Mariusz Florek, Maciej Bąkowski, Grzegorz Skiba

**Affiliations:** 1 Institute of Animal Nutrition and Bromatology, University of Life Sciences in Lublin, Akademicka St. 13, 20-950 Lublin, Poland; eugeniusz.grela@up.lublin.pl (E.R.G.); maciej.bakowski@up.lublin.pl (M.B.); 2 Department of Animal Nutrition and Feed Science, National Research Institute of Animal Production, Krakowska St. 1, 32-083 Balice, Poland; 3 Department of Quality Evaluation and Processing of Animal Products, University of Life Sciences in Lublin, ul. Akademicka 13, 20-950 Lublin, Poland; mariusz.florek@up.lublin.pl; 4 The Kielanowski Institute of Animal Physiology and Nutrition Polish Academy of Sciences Instytucka 3, 05-110 Jabłonna, Poland; g.skiba@ifzz.pl

**Keywords:** prebiotics, inulin, artichoke, probiotic bacteria, meat quality, physicochemical traits, sensory properties, dietary value, fatty acid composition, pigs

## Abstract

**Simple Summary:**

Fructooligosaccharides, including inulin, are prebiotics involved in the regulation of bacterial flora, intestinal health, and metabolism in animals. Oligosaccharides and inulin-containing plants are the subject of numerous studies around the world. Inulin is an important oligosaccharide, considered a prebiotic in the diet of humans and animals. In this study, we examined the effect of the supplementation of probiotic bacteria in the diet with inulin or dried Jerusalem artichoke tubers on the performance, pig meat quality, and fatty acid profile of meat and backfat, which provided novel information on the use of these additives in livestock production. An improvement in the antioxidant status of meat and in the water-holding capacity, as well as a reduction in the shear force after the addition of both prebiotics can be mentioned as the pertinent results. However, the meat sensory traits were unchanged by supplementation with the prebiotics and probiotics. This study forms part of the current global work on feed additives that support animals’ health and concurrently allow the use of antibiotic treatments to be limited. Therefore, the results of this experiment are of great practical importance and can be proposed for use in pig farming as part of the intensive work which is underway across the EU to reduce the use of antibiotics. In addition, meat with higher quality and a longer shelf life derived from pigs fed with feed containing additives of natural origin are sought by consumers who value healthy and safe food.

**Abstract:**

In this experiment, we investigated the effect of the supplementation of probiotic bacteria in the diet with inulin or dried Jerusalem artichoke tubers on the performance, meat quality, and fatty acid composition in the meat and backfat of fatteners. One hundred and forty-four crossbred pigs (PIC × Penarlan P76) were divided into six groups and fattened from 30 to 114 kg. The meat proximate composition, pH, color, texture, shear force, water-holding capacity, sensory attributes, and thiobarbituric-acid-reactive substances were measured. Normal post-mortem meat glycolysis was demonstrated and no meat defects were present. The chemical constituents in muscle tissues were similar, except for intramuscular fat (IMF). The addition of the prebiotics resulted in a higher IMF level, whereas a significantly lower content was found after the probiotic supplementation. Meat from both prebiotic groups was lighter, less red, and more yellow and showed a higher hue angle. The addition of both prebiotics significantly improved the antioxidant status of meat (by approximately 16% and 18%) and the water-holding capacity (less free water and higher M/T ratios), but reduced shear force (by 17%, *p* ≤ 0.05) and hardness (by 39% and 35%, respectively, *p* ≤ 0.05). The addition of the prebiotics and probiotics had no effect on any of the evaluated sensory attributes.

## 1. Introduction

In the post-antibiotic era, it is important to have a complete understanding of how gut bacteria grow and interact with prebiotics and with animals. Probiotics and prebiotics are among many additives considered for use in pig nutrition that deserve attention. Various types of oligosaccharides, e.g., inulin, are regarded as a source of prebiotics. Inulin is a water-soluble fructose polymer found mainly in chicory roots (*Cichorium intybus*) or Jerusalem artichoke tubers (*Helianthus tuberosus*). It contains both oligosaccharides and polysaccharides, which are responsible for its prebiotic properties [1]. Inulin is composed of a glucose molecule linked with a chain of fructose molecules (from a few to several tens) [2]. It is included among the prebiotics, i.e., substances promoting the growth of bacteria that constitute the normal intestinal flora [3]. Dietary inulin and FOS are not hydrolyzed by mammalian enzymes, but are readily fermented by the bacterial community in the caecum and colon and favor the growth of intestinal bifidobacteria [4]. The growth of *Bifidobacteria* and *Lactobacillus* is the best-known effect of inulin [5], which exerts a health-enhancing impact through the stimulation of these bacteria [6,7]. The microflora produce numerous nutraceutical compounds, e.g., organic acids, hydrogen peroxide, acidolin, acidolphillin, reuterin, lysozyme, lactoferrin, and lactoperoxidase, as well as such bacteriocins such as lactocidin and lactocin, which exhibit immunomodulatory and antibacterial properties [8,9]. Through the promotion of the growth of lactic acid bacteria, inulin exerts an indirect beneficial effect on the host immune system through, e.g., the production of anti-inflammatory cytokines, mononuclear cells, and phagocytic macrophages. It is also involved in the induction of the synthesis of immunoglobulins, in particular IgA [10]. Inulin has a positive effect on the intestinal immune system, blood flow through the mucosa, and the activity of the local nervous system. Additionally, it increases the intestinal absorption of minerals (improved absorption of iron, zinc, magnesium, and calcium ions in in vitro studies). It also modulates insulin and sugar levels, plasma lipids (cholesterol and triglycerides), weight reduction, and, secondarily, the course and development of atherosclerosis and its complications. Pigs are an excellent model with regard to human metabolism. Hence, the obtained results concerning, among other things, the metabolic profile of blood, as well as the composition of fatty acids deposited in the muscles and reserve fat of pigs, may provide an interesting reference as to the effectiveness of the use of probiotics and prebiotics in the human diet.

The aim of this study was to assess the effect of the addition of a probiotic composed of the following bacterial strains: *Lactococcus lactis* IBB500 min > 109 cfu/g, *Carnobacterium divergens* S1 min > 109 cfu/g, *Lactobacillus casei* ŁOCK 0915 min > 109 cfu/g, *Lactobacillus plantarum* ŁOCK 0862 min > 109 cfu/g, and *Sacharomyces cerevisiae* ŁOCK 0141 min > 107 cfu/g to diets supplemented with pure inulin (linear β fructans with a degree of polymerization (DP) ≥ 15) or inulin contained in dried Jerusalem artichokes on the production performance, post-mortem carcass quality, technological parameters of meat, and the composition of fatty acids in backfat and the *longissimus lumborum* muscle.

## 2. Materials and Methods

### 2.1. Ethical Approval

Ethical Review and Approval was waived for this study, as according to Polish law, Ethical Approval is not required for services within the scope of the Act of 18 December 2003 on animal treatment facilities, as well as agricultural activities, including the rearing or breeding of animals, carried out in accordance with the provisions on the protection of animals, and activities that, in compliance with the practices of veterinary medicine, do not cause pain, suffering, distress, or permanent damage to the body of animals, to an extent equal to or more intense than a needle prick (Act of the Protection of Animals Used for Scientific and Educational Purposes, Legislative Decree 266/2015). The experiment was conducted according to the guidelines of the Declaration of Helsinki and in compliance with the European Union law (Directive 2010/63/UE, received in Poland by Legislative Decree 266/2015) of the European Parliament and of the council on the protection of animals used for scientific or educational purposes.

### 2.2. Animals, Housing, and Treatment

The experiment involved 144 crossbred gilts and barrows, originating from PIC hybrid line sows mated with Penarlan P76 boars. The initial body weight was 30.0 ± 0.5 kg. The pigs were randomly assigned to 6 groups, with 24 pigs (12 gilts and 12 barrows) in each group. Pigs in group I received standard (control) diets in both fattening periods, i.e., grower and finisher diets. Group II received 20 g/kg of inulin, and the diets for group III contained 40 g/kg of dried Jerusalem artichoke instead of maize starch. Based on the research of other authors [1] and our own experiments, it is considered advisable to use 20 g/kg of inulin with a high degree of polymerization (DP > 15), whereas the addition of Jerusalem artichoke in an amount of 40 g/kg was based on an approximately 50% inulin content in the feed (30–70%) [1,11,12]. A probiotic was added in the diets in amount of 1 g/kg diets for animals in groups IV, V, and VI, in addition to the experimental factor analogous to groups I, II, and III (Table 1).

The pigs in each group were kept in pens: 8 pens per group with 3 pigs in each pen. The pens were separated by sex (gilts were kept separately from the barrows). The fatteners were fed a grower mixture (30–70 kg BW) and a finisher mixture (71–114 kg BW). The body weight was controlled for individual animals. The basal diets comprised ground grain (wheat and barley), corn starch, soybean meal, soybean oil, minerals (salt, monocalcium phosphate, and ground limestone), and a mineral-vitamin premix (Table 2).

The diets were balanced for metabolizable energy, total protein, amino acid composition, minerals, and vitamins according to NRC [14]. All the animals had ad libitum access to feed and water. The hygienic conditions, i.e., temperature, relative humidity, and cooling, were optimal for the fattened pigs and were the same for all the groups. During the experiment, each animal was weighed 3 times (at the beginning, at 70 kg BW, and before slaughter). The feed intake was controlled in the individual pens by weighing the portions for automatic feeding in the cages. All pigs were slaughtered after 98 days of fattening.

The requirements for the road vehicle and conditions of animal transport were in conformity with the applicable European Union rules [15]. Each transport started in the morning (6:00–7:00 a.m.). The distance from the farm to the slaughterhouse was about 50 km and, after 12-h fasting, the animals were slaughtered in accordance with the provisions of Council Regulation No 1099/2009 [16]. The carcasses were chilled and cut in accordance with the standard technology employed in the meat industry and under official veterinary supervision. The content of lean meat in the carcass was measured according to the method proposed by Różycki and Tyra (2010) [17] after 24-h chilling at 4 °C. The backfat thickness was measured with a vernier caliper (accuracy of 0.1 mm) on the right half-carcasses at 5 points: over the shoulder, on the midback, and on the rump at three points (over the cranial, medial, and caudal edge of the gluteus medius muscle cross-section). Longissimus lumborum muscle samples were taken for analysis near the last thoracic and first lumbar vertebrae, whereas the backfat samples were collected over the shoulder blade, cutting out a lobe of 5 cm in width and 10 cm in length from a forequarter cut. Immediately after collection, the samples were stored at −20 °C. The meat and backfat samples from 8 animals (4 gilts and 4 barrows) per group, with a body weight close to the average body weight in the group, were taken for analysis. The muscle samples for sensory analysis, weighing around 500 g, were vacuum packed and stored at 2 °C up to 96 h post-slaughter.

### 2.3. Analysis of Feeds 

The diet samples were analyzed for their contents of basic nutrients, according to standard AOAC procedures [18]. The calcium content was determined in an ASA SOLAR 939 UNICAM flame spectrophotometer, whereas the phosphorus content was assessed with the spectrometric method, according to the AOAC [18]. Total lysine was determined with ion-exchange chromatography in a 119 Cl Beckman amino acid analyzer (Beckman Instrument Company, Brea, CA, USA). Prior to the analyses, the samples were subjected to acidic hydrolysis in the presence of 6 M HCl at 105 °C for 24 h. Sulfuric amino acids were determined separately after oxidation [19,20]. The fatty acid content in the feed fat was determined by means of gas chromatography.

### 2.4. Meat Analysis

Physicochemical meat properties were determined in raw samples of the m. longissimus lumborum according to the methodology described previously [21]. Meat pH was measured 45 min and 24 h after slaughter using a penetrating glass electrode ERH-12-6 (HYDROMET, Gliwice, Poland) and a CP-401 portable pH-meter (Elmetron, Zabrze, Poland) equipped with a temperature sensor. The pH electrode was calibrated in 2 points (pH 4.00 and pH 7.00) according to the manufacturer’s instructions with high-accuracy (±0.02 at 20 °C) pH buffer solutions (Elmetron, Zabrze, Poland). The pH-meter allowed the automatic detection of buffer solutions and carried out automatic compensations for temperature. Meat color parameters according to the CIE L*a*b* system [22] were measured using a Minolta CR-310 color saturation meter (illumination/projection D65/10°) on the muscle surface after blooming (30 min) in refrigerated conditions (4 °C ± 1 °C). Each color measurement was conducted three times on the surface of each meat sample, and the mean value of these measurements was taken as the result for a given sample.

The TBARS value (2-thiobarbituric acid reactive substances) was determined according to the method developed by Witte et al. [23] using a Varian Cary 300 Bio spectrophotometer (Varian Australia PTY, Ltd., Mulgrave, Australia) at 530 nm. The TBARS values were calculated by multiplying absorbance by 5.2, and the results were expressed as mg of malondialdehyde (MDA) per kg of muscle tissue.

The filter paper press method [24] was used to measure the amount of expressible water (mg) from the meat (300 mg) held under pressure (2 kg for 5 min). The total liquid infiltrated area (T) and meat spots (M) were measured (cm^2^) using imaging software (MultiScan Base ver. 14), and the proportion of M/T × 100 [25] was calculated. Drip loss was determined as a percentage based on the difference in the weight of the sample before and after storage at 4 °C for 24 h. Cooking loss was determined based on the difference in the weight of the muscle samples (100 ± 10 g) before and after heat treatment at 70 °C for 60 min in a water bath.

The analyses of cooked samples (after the determination of cooking loss) included measurements of color coordinates (in the CIE L*a*b* system) and texture parameters. Shear force (N) and shear energy (J) measurement was carried out using a Zwick/Roell testing machine Proline BDO125 FB0.5TS (Zwick GmbH & Co, Ulm, Germany) and Warner–Bratzler device (V-blade). For the texture profile analysis (TPA), the Zwick/Roell Proline machine was fitted with a 70-mm-diameter compression plate to determine the hardness, springiness, gumminess, and chewiness of the meat samples. Each measurement was conducted in three replications, and the results were presented as mean values of these replications using TestXpert® II software [26].

### 2.5. Fatty Acid Analysis

Total fat was extracted from the backfat and m. lumborum muscle for fatty acid analysis with a chloroform/methanol mixture (2:1 *v/v*) according to the method proposed by Folch et al. [27]. Fat from the diets was extracted with hexane [18]. Further investigations of the fatty acid profile were conducted according to the standards PN-EN ISO 12966-2:2017-05 [28] and PN-EN ISO 12966-1:2015-01 [29]. Fatty acids were analyzed as methyl esters using the gas chromatography procedure on a Varian CP-3800 chromatograph (Varian INC, Palo Alto, CA, USA). Fatty acids were saponified (0.5 N NaOH in methanol, 80 °C) and then esterified with boron trifluoride/methanol according to ISO 12966-2:2017 [30]. After extraction with hexane, the compounds were separated on the column using a flame ionization detector and AOC-20i autosampler (Shimadzu, Kyoto, Japan). The extraction efficiency was 97.1% on average. The chromatograph operating conditions for fatty acid separation were as follows: capillary column CP WAX 52CB DF 0.32 mm of 105 m length, gas carrier—helium, flow rate 1.4 mL/min, column temperature 120 °C, gradually increasing by 2 °C/min up to 210 °C, determination time 135 min, feeder temperature 160 °C, detector temperature 160 °C, other gases—hydrogen and oxygen. Fatty acids were calculated using chromatogram peak areas and expressed as g/100 g fatty acid methyl esters [31].

### 2.6. Lipid Quality Indices

Lipid quality indices, i.e., the atherogenicity index (AI) and the thrombogenicity index (TI) were calculated according to the equations formulated by Ulbricht and Southgate [32]:

AI = [C12:0 + (4 × C14:0) + C16:0]/[n-6 PUFA + n-3 PUFA + MUFA]
(1)

TI = [C14:0 + C16:0 + C18:0]/[(0.5 × MUFA) + (0.5 × n-6 PUFA) + (3 × n-3 PUFA) + n3/n-6 PUFA]
(2)

The hypocholesterolemic/hypercholesterolemic ratio (h/H) was calculated according to the formula proposed by Fernández et al. [33]:

h/H = (C18:1 + C18:2 + C18:3 + C20:3 + C20:4 + C20:5 + C22:4 + C22:5 + C22:6)/(C14:0 + C16:0)
(3)

### 2.7. Sensory Analysis

The sensory evaluation was performed 4 times using a total number of 12 samples of longissimus lumborum muscle. Meat samples were cooked with a salt solution (0.8% NaCl) [34] in a water bath at 80 °C to reach the endpoint temperature of 72 °C, monitored with thermocouples placed in the geometric center. Samples were prepared, coded, and presented to panelists as described by Greguła-Kania et al. [35]. The sensory evaluation was conducted by an 8-member trained panel (4–7 years of sensory evaluation practices) according to PN-ISO 4121:1998 [36]. Briefly, the samples were cut into representative cubes and evaluated for the color, odor, palatability, and consistency. The panelists were served two random cubes of each sample, assigned a 3-digit blind code. A five-point scale (1—unacceptable, 2—poor, 3—satisfactory, 4—good, 5—very good) was applied.

### 2.8. Statistical Analysis

Data were analyzed in a split plot designed using the Statistica software, version 13.1 (Dell Inc. 2016, Round Rock, TX, USA) [37]. The data were checked for normality and homogeneity of variance by means of the Shapiro–Wilk and Brown–Forsythe tests, respectively. The pen served as the experimental unit for feed intake and feed conversion ratio (n = 8 per group), whereas the individual pig served as the experimental unit for body weight, average daily gain, carcass traits, and fat and fatty acid composition in tissues (n = 24 per group). All meat quality indices and fatty acid composition in tissues were analyzed for 8 pigs (4 gilts and 4 barrows per group).

The obtained data were analyzed statistically using a general linear model (GLM) of the two-way ANOVA. Tukey’s test was applied for multiple comparisons among the means. Differences were considered significant at a level of *p* < 0.05. No significant interactions between treatments and gender were found; therefore, the data were pooled according to gender in this paper (*p* > 0.05). The model included the fixed effects of dietary treatment, gender (gilts/barrows) and the associated interaction, and block as a random effect. The tables illustrate the means, the standard error of means (SEM), and the levels of significance in the main factors (Pre, Pro) and interactions (*p*-value).

## 3. Results

The chosen indices of fattening performance and carcass slaughter analysis are presented in Table 3. The probiotic and prebiotic supplementation improved the average body weight gains (*p* ≤ 0.05); however, in the case of the prebiotic factor, a significant effect was noticed in the group receiving inulin. None of the additives affected the feed conversion ratio. The prebiotic influenced the backfat thickness, which was lower in pigs fed with dried Jerusalem artichoke (*p* ≤ 0.05). Neither the prebiotic nor probiotic used in this experiment affected the weight of the analyzed organs (liver and kidneys).

The results of proximate composition, TBARS content, and pH values of the m. longissimus lumborum of fatteners fed with prebiotics and probiotics are presented in Table 4. In general, the addition of the prebiotics resulted in a higher percentage of intramuscular fat (IMF); however, the significantly highest level was found in the DJA-40 group, in comparison with the I-20 and C-0 groups. In contrast, the addition of the probiotics (Pro+) reduced its content significantly (*p* ≤ 0.05) in the longissimus lumborum muscle compared to the fatteners from the Pro− group. The addition of both prebiotics (I-20 and DJA-40) significantly (*p* ≤ 0.05) improved (by approximately 16% and 18%) the antioxidant stability of meat, as expressed by the TBARS value. The meat pH was not affected by any feed additives and their interaction. The initial and ultimate meat pH ranged from 6.57 to 6.64 and from 5.69 to 5.72, respectively. However, lower pH_45_ values (6.57–6.59) were found in meat from the I-20 and DJA-40 groups in comparison with the C-0 group (6.64).

The addition of I-20 significantly (*p* ≤ 0.05) modified all instrumentally measured color parameters (except L*) compared to the C-0 group, but only in fresh meat (Table 5). The addition of DJA-40 had a similar effect. Fresh meat from both prebiotic groups was brighter, less red, and more yellow and showed a higher hue angle. In contrast, the color of meat from fatteners fed with the probiotics (Pro+) did not differ from that of pigs from the Pro− group. The heat treatment of the meat (denaturation of muscle proteins) eliminated all differences in the color indices between the groups. 

The addition of the prebiotics and probiotics had no effect on any of the evaluated sensory attributes, and no interaction was found between these factors (Table 6). However, scores higher by 0.2 pts were obtained for the palatability of the meat from fatteners receiving both prebiotics (I-20 and DJA-40 vs. C-0) and probiotics (Pro+ vs. Pro−). No significant interaction was found for the assessed texture parameters of cooked meat, except for WB shear force (SF WB) (Table 6). As for the main effects of the individual factors, only the effect of the prebiotic on shear force and shear energy in the Warner–Bratzler test, as well as hardness, gumminess, and chewiness in the texture profile analysis (TPA) test, were found to be significant (Table 6). The addition of inulin, irrespective of its source (I-20 or DJA-40), significantly (*p* ≤ 0.05) improved all meat texture characteristics (except springiness) compared to the control group (C-0) (Table 6). The addition of inulin (I-20 and DJA-40) reduced WB shear force by 17% (*p* ≤ 0.05) and 6%, and energy force by 27% (*p* ≤ 0.05) and 23% (*p* ≤ 0.05), respectively. In the TPA test, hardness, gumminess, and chewiness decreased significantly (*p* ≤ 0.05) by 39%, 34%, and 35% in the I-20 group and by 35%, 21%, and 21% in the DJA-40 group, compared to the C-0 group. The extent of the improvement was significantly greater for the addition of I-20 than that of DJA-40 with regard to gumminess and chewiness. In contrast, the diet supplemented with the probiotics had no effect on the textural parameters of meat.

More favorable values of the water-holding capacity parameters, including the significantly (*p* ≤ 0.05) lower amounts of free water and the higher M/T ratios, were found for the meat samples of fatteners fed with the inulin additive (I-20 and DJA-40) compared to the control group (Table 7). The addition of the probiotics to the feed did not affect the water-holding capacity of the meat.

The fatty acid composition of meat is presented in Table 8. The prebiotic supplementation did not influence the fatty acid content in the meat, except C16:1, which was increased by the dried Jerusalem artichoke as well as the PUFA n-6/n-3 ratio, which was decreased by this prebiotic (*p* ≤ 0.05). The probiotic additive in feed significantly lowered the C18:2 fatty acid content in the meat (by 7.2%; *p* ≤ 0.05), which resulted in an approx. 6% lower PUFA n-6 and PUFA n-6/n-3 ratio. The atherogenicity and thrombogenicity indexes and the h/H ratio were not affected either by the pre- or probiotic supplement (Table 8).

In the case of the backfat, the dried Jerusalem artichoke decreased the SFA content (*p* ≤ 0.05) in comparison to the control group, mostly because it reduced the content of C14:0 fatty acid by about 9% (Table 9). Both prebiotics lowered the thrombogenicity index and increased the h/H ratio, but a significant difference was observed only between the dried Jerusalem artichoke and the control group (*p* ≤ 0.05). There was no significant difference in the fatty acid composition in the backfat between pigs fed with or without the probiotic supplement.

## 4. Discussion

Genetic factors and rearing conditions, including nutrition, determine the efficiency of pig rearing and the production of high-quality pork [38]. The content and quality of nutrients, mainly protein and energy, largely determine the nutritional and dietary value of pork [39]. Feed additives are also noteworthy, as they shape the microbiome of the digestive tract, influence the health of animals, and improve the utilization of nutrients. Due to the significant or complete limitation of the use of antibiotic growth promoters (AGP), phytobiotics, eubiotics, probiotics, and prebiotics are increasingly being used. Previous studies have shown the beneficial effects of oligosaccharides [40] and probiotic bacteria [41], as well as synbiotics [42] in piglets and fatteners. The enrichment of diets with these additives has contributed to the reduction of diarrhea and better weight gain in piglets [43]. In fatteners, it improved the digestibility and utilization of feed nutrients and the quality of pork [44].

After 98 days of fattening, the average body weight of fatteners was 114.2 kg. The differences in the individual fattening periods were similar to those for the whole fattening period, so we did not provide values for individual fattening periods. The results of the present study showed higher final weights and better daily gains when the pigs were fed with diets supplemented with both inulin and with dried Jerusalem artichokes; however, a stronger effect was observed in the case of inulin supplementation. This resulted from the better action of chemically pure inulin than inulin contained in the dried Jerusalem artichoke. The additional enrichment of the feed with the probiotic increased this effect and slightly improved feed conversion. Recent studies have highlighted the suitability of inulin in pig nutrition. The most visible is its beneficial effect on growth performance in growing–finishing pigs [45], mainly via a positive effect on the duodenum and ileum morphology (the ratio of villus height:crypt depth); elevated activity of sucrose, glucose, and metal transporters in the ileum mucosa; decreased pathogen bacteria; and the expression of pro-inflammatory cytokines [46].

The additives used in the present study also contributed to the differentiation of the fat content in the pig carcasses, as the animals fed with diets supplemented with both forms of the prebiotics had lower backfat thicknesses at all measuring points. It is likely that the oligosaccharides contained in Jerusalem artichokes, as well as in pure inulin, limited the deposition of stored fat via the production of short-chain organic acids in the large intestine. As reported by Birmani et al. [47], the explanation is that the inulin oligosaccharide can reduce the expression and activity of fat-producing enzymes in the liver, consequently reducing the synthesis of fatty acids and triglycerides.

The measurement of muscle tissue pH allows the identification of quality defects in raw pig meat, most often as PSE or acidic meat. The typical meat pH obtained as a result of post-slaughter glycogenolysis ensures favorable sensory and technological properties, including an attractive color, tenderness, and palatability, as well as a good water-holding capacity [48]. It is assumed that an ultimate pH (24 and 48 h after slaughter) of case-ready pig meat should have a range of 5.50 to 5.80 [49]. In the present study, the ultimate pH of the LL muscle ranged from 5.69 to 5.72, indicating correct handling of the animals (good welfare) before slaughter. The risk of meat defects (RSE or PSE) is minimal at an ultimate pH >5.7 [50]; therefore, a higher ultimate pH should improve meat quality. Despite the observed differences, the TBARS value was at a relatively low level, since odor deviations in pig meat are perceptible sensorially in the range of 0.5 to 1.0 mg MDA/kg meat [51]. The higher oxidative stability (lower TBARS values) found in the muscles of fatteners receiving the addition of the prebiotics (I-20 and DJA-40) may be related to the lower content of muscle pigments (myoglobin), which show pro-oxidative effects due to the presence of Fe (according to Min et al. 2008) [52]. This is indicated by higher lightness (higher L* values) and lower redness (lower a* value), which are correlated with heme pigments in the meat of pigs (according to Lindahl et al., 2001) [53]. Furthermore, it should be noted that the higher antioxidant stability was not associated with the higher IMF content in fatteners fed with the prebiotics (I-20, *p* ≤ 0.05; DJA-40, *p* > 0.05). The exact cause of these differences is difficult to explain unequivocally, as literature data on the effect of inulin and probiotic administration on the antioxidant status of pork are not known. However, Herosimczyk et al. [54] showed that pigs fed with a diet supplemented with 1% or 3% native chicory inulin had significantly reduced liver TBARS levels (*p* < 0.10) compared to the control group. Hansen et al. (2008) [55] fed pigs before slaughter with a feed rich in fermentable fiber (10–13.3% dried chicory roots) and reported values similar to those presented in this study for LD muscle parameters such as lightness (L* = 57.09) and drip loss (3.83%) but reported a lower value of ultimate pH (5.61).

The sensory impression is of great importance for the acceptability and quality of meat products. Therefore, meat producers/animal breeders are interested in optimal solutions that can be used to obtain high-quality products. In this context, it is of interest to be able to immediately influence the sensory quality of pork meat through the simple manipulation of feed ingredients, including fructooligosaccharides/inulin [56]. However, the final decision to use inulin from chicory depends on the feeding period, the sex of the pigs, and the expected and desired sensory properties of the meat [55]. In the present study, the meat from gilts and barrows receiving prebiotic and probiotic supplementation in their diet was evaluated. Fermentable carbohydrates, such as inulin, are most commonly used in nutrition for boars (uncastrated males) [57,58], due to the effective reduction of the skatole concentration in the hindgut and adipose tissue, thus reducing the incidence of boar taint [21]. Byrne et al. (2008) [59] showed that raw and dried chicory and inulin did not lead to a new negative taste and odor sensory characteristics in cooked pork. Even the bitter taste notes characteristic of chicory roots did not cause a negative overall impression. In the case of the effect of inulin on the sensory profile of pork, it was found to be unique, expressed in terms such as feedy, umami, and parsnip. Moreover, there is no explanation for this phenomenon in the literature.

The tenderness of the meat after cooking, expressed as the shear force in the WB test, was significantly different between fatteners fed with or without the prebiotics (Table 6). The significantly (*p* ≤ 0.05) highest values of WB SF were found for the meat of fatteners from the control group (C-0) (64 N on average), whereas the lowest values were obtained in the meat of fatteners fed with the I-20 addition (53.35 N on average). Taking into account the different courses of aging of pig meat, Iwańska et al. (2016) [60] proposed the following classification of meat tenderness according to WB SF (N/cm^2^): very tender < 30, tender 30–45 N, tough 60–90 N, and very tough > 90 N. Assuming this division into classes in the present study, the meat from the control group can be classified as tough and that of the experimental groups (I-20, DJA-40, and Pro+) as intermediate (between tender and tough). However, it should be stressed that these levels of tenderness were already obtained 48 h after slaughter and the meat was not subjected to the aging process.

Rosenvold et al. (2001) [61] investigated the effect of a high inulin supplement (25%) feeding for 4 weeks before slaughter on the slaughter value and meat quality of gilts. This diet resulted in significantly lower drip loss (4.0%, *p* < 0.05) and higher WB shear force of the LD muscle (43 N, *p* < 0.05) compared to gilts from the control group (5.2% and 38.5 N, respectively). However, ultimate pH, color (CIE L*a*b*), and cooking losses were not affected. The researchers proposed that the reduced tenderness was probably related to reduced muscle glycogen stores, resulting from a feed that contained low levels of digestible carbohydrates and high levels of fermentable carbohydrates. On the contrary, Aluwé et al. (2013) [62] showed no effect of inulin addition (from dried pulp and dried chicory roots) on LD muscle quality parameters, except for higher drip loss.

Recently, in regard to the fatty acids analyzed out in our research, we have paid attention to the muscle tissues, because of their greater importance in food in comparison to backfat, and due to consumers’ interest in the dietary quality of meat. The present study shows that supplementation of a diet with a prebiotic (inulin) and a probiotic can influence the fatty acid composition and health-promoting indices of pork products. However, the obtained results were far from the values recommended by the WHO. The literature data concerning the influence of the supplementation of diets with inulin and probiotics on the tissue fatty acid composition are ambiguous. In a study conducted by Brestenský et al. (2016) [63], the addition of a mixture of inulin and horse chestnuts had no effect on the fatty acid profile, including the ratio of PUFAs n-6/n-3, in the longissimus dorsi muscle of pigs. In a study conducted by Chang et al. (2018) [64], the meat of the probiotic-supplemented group (*Lactobacillus plantarum*) showed higher PUFA contents, with significantly higher levels of linolenic and linoleic acid compared to the control group. Similarly to our results, the PUFA n-6/n-3 ratio was also higher than the values recommended for human health. According to results reported by Juárez-Silva et al. (2019)[65], the addition of inulin into rabbits’ diets increased the content of beneficial fatty acids (CLA and n3-PUFA) and ensured a better health-promoting index, while reducing the atherogenic and thrombogenic indices of the meat. Similarly, Grela et al. (2014) [66] found that the fatty acid composition and AI, TI, and h/H indexes of the backfat and meat of fatteners fed with a prebiotic-enriched diet (dried inulin-rich dandelion) indicated a reduced risk of developing atherosclerotic disorders. Contrarily, data presented by Mattioli et al. (2017) [67] showed that dietary supplementation with prebiotic compounds (inactivated *S. cerevisiae* yeast) did not enhance the bioactive fatty acid content in rabbit meat.

## 5. Conclusions

This study showed no significant effect of the addition of probiotics on the physicochemical properties and sensory attributes of pig longissimus lumborum muscles. There was also no significant prebiotics × probiotics interaction in the assessed parameters of pork quality. The results of the present study indicate that the addition of inulin from different sources as prebiotics to pig feed does not have a negative effect on the physicochemical properties of meat and demonstrate its potential technological suitability for further processing and case-ready purposes. The addition of the prebiotics was found to increase the intramuscular fat content; however, a reduced content of IMF was observed in the meat from pigs fed with the probiotics. Regardless of its source, inulin supplementation beneficially influenced antioxidant stability, water-holding capacity, and the tenderness of meat. On the basis of the obtained results, it can be concluded that prebiotic supplementation of both 20 g/kg of inulin and 40 g/kg of dried Jerusalem artichoke, into the diet of finishing pigs, can be used as a feed additive, improving the quality properties of meat.

## Figures and Tables

**Table 1:** Experimental design.

Groups	I	II	III	IV	V	VI
Prebiotic supplement (Pre), g/kg *	0	20 (I)	40 (DJA)	0	20 (I)	40 (DJA)
Probiotic supplement (Pro) **	-	-	-	+	+	+

* Prebiotic: (I) inulin-linear β fructans with a degree of polymerization ≥ 15; (DJA) dried Jerusalem artichoke. ** Probiotic: bacterial strains *Lactococcus lactis* IBB500 min > 10^9^ cfu/g, *Carnobacterium divergens* S1 min > 10^9^ cfu/g, *Lactobacillus casei* ŁOCK 0915 min > 10^9^ cfu/g, *Lactobacillus plantarum* ŁOCK 0862 min > 10^9^ cfu/g, *Sacharomyces cerevisiae* ŁOCK 0141 min > 10^7^ cfu/g.

**Table 2:** Composition (g/kg) and nutritive value of growing–finishing pig diets.

Item	Grower Phase			Finisher Phase		
	I, IV	II, V	III, VI	I, IV	II, V	III, VI
Wheat	300	300	300	300	300	300
Barley	375	375	375	479	479	479
Corn starch	40	20	-	40	20	-
Soybean meal	242	242	242	150	150	150
Soya oil	12	12	12	-	-	-
Limestone	7	7	7	7	7	7
Monocalcium phosphate	4	4	4	4	4	4
Mineral-vitamin premix *	20	20	20	20	20	20
Inulin (DP ≥ 15)	-	20	-	-	20	-
Dried Jerusalem artichoke	-	-	40	-	-	40
Content in 1 kg of feed mixture (analyzed values):						
Dry matter, g	898.6	897.2	897.5	889.2	890.1	892.5
Crude ash, g	45.91	45.89	45.93	41.64	41.58	41.69
Crude protein, g	170.4	170.2	170.3	153.2	152.5	151.8
Ether extract, g	38.65	38.61	38.68	32.6	32.2	32.5
Crude fibre, g	46.8	46.4	46.1	44.7	44.4	43.8
EM, MJ **	12.93	12.86	12.58	12.64	12.53	12.38
Total lysine, g	10.76	10.75	10.77	8.81	8.78	8.79
Methionine + cysteine, g	5.82	5.79	5.83	5.40	5.39	5.41
Calcium, g	7.10	7.11	7.13	6.48	6.44	6.46
Total phosphorus, g	5.07	5.05	5.09	4.87	4.88	4.85
Natrium, g	2.01	2.01	2.04	2.05	2.01	2.03
Palmitic acid (16:0), g	2.08	2.04	2.05	2.27	2.25	2.26
Stearic acid (18:0), g	0.26	0.24	0.25	0.23	0.22	0.21
Oleic acid (18:1), g	2.61	2.59	2.63	2.48	2.44	2.51
Linoleic acid (18:2), g	6.55	6.62	6.48	6.69	6.71	6.64
Linolenic acid (18:3), g	1.04	1.01	1.03	1.03	1.01	1.02

* 1 kg of the mineral-vitamin premix contained vitamin A 600,000 i.u., D_3_ 60,000 i.u., E 3000 mg, K_3_ 120 mg, B_1_ 120 mg, B_2_ 240 mg, B_6_ 240 mg, nicotinic acid 1600 mg, pantothenic acid 800 mg, folic acid 160 mg, biotin 10 mg, B_12_ 1.6 mg, choline chloride 12 g, Mg 0.8 g, Fe 6 g, Zn 5.6 g, Mn 2.4 g, Cu 6.4 g, J 40 mg, Se 16 mg, and Co 16 mg. ** Metabolizable energy was calculated according to the equation proposed by Kirchgessner and Roth [13].

**Table 3:** Selected indicators of fattening and slaughter analysis of carcasses.

Item	Prebiotic Supplement (Pre)			Probiotic Supplement (Pro)		SEM	*p*-Value		
	Control 0	Inulin 20	DJA 40	−	+		Pre	Pro	Interaction
ADG (30–114 kg), g	844 ^b^	863 ^a^	854 ^ab^	843 ^b^	864 ^a^	19.1	0.048	0.047	0.031
FCR (30–114 kg), kg/kg	2.73	2.65	2.70	2.73	2.65	0.15	0.096	0.094	0.106
Final body weight after 98 days of fattening, kg	113.5 ^b^	115.1 ^a^	114.1 ^ab^	113.5	114.9	1.09	0.047	0.056	0.097
Cold dressing, %	79.0	78.3	78.2	78.7	78.3	0.57	0.109	0.112	0.053
Carcass lean meat content, %	55.0	55.8	55.5	54.9	55.9	0.62	0.208	0.095	0.048
Loin eye, cm^2^	54.0	54.5	54.4	53.8	54.8	0.54	0.189	0.107	0.041
Backfat thickness, mm									
-shoulder	31.3 ^a^	29.1 ^b^	30.1 ^ab^	30.7	29.5	0.14	0.041	0.064	0.051
-midback	21.8 ^a^	21.6 ^a^	19.8 ^b^	21.2	20.8	0.12	0.048	0.108	0.145
-rump (3 measurements)	22.2 ^a^	22.6 ^a^	20.9 ^b^	22.2	21.5	0.13	0.046	0.102	0.095
-average from 5 measurements	23.9 ^a^	23.7 ^a^	22.5 ^b^	23.7	23.0	0.19	0.047	0.093	0.084
Weight of kidney, g	164.4	165.4	165.9	165.3	165.2	7.83	0.605	0.859	0.467
Weight of liver, kg	1.80	1.82	1.83	1.82	1.81	0.14	0.408	0.592	0.394

^a, b^—statistically significant differences at *p* ≤ 0.05; ADG—average daily weight gains; FCR—feed conversion ratio.

**Table 4:** Proximal composition, TBARS, and pH of meat (m. longissimus lumborum).

Item	Prebiotic Supplement (Pre)			Probiotic Supplement (Pro)		SEM	*p*-Value		
	Control 0	Inulin 20	DJA 40	−	+		Pre	Pro	Interaction
Moisture, %	71.65	71.41	70.86	71.47	71.14	0.55	0.298	0.594	0.288
Protein, %	24.98	25.15	25.37	24.93	25.40	0.53	0.386	0.237	0.396
Fat, %	2.09 ^b^	2.13 ^b^	2.26 ^a^	2.22 ^a^	2.09 ^b^	0.15	0.046	0.044	0.163
Ash, %	1.14	1.14	1.17	1.13	1.16	0.02	0.394	0.283	0.231
TBARS, mg MDA/kg meat	0.38 ^a^	0.32 ^b^	0.31 ^b^	0.34	0.33	0.01	0.043	0.354	0.404
pH_45_	6.64	6.57	6.59	6.60	6.59	0.10	0.324	0.592	0.306
pH_24_	5.72	5.70	5.70	5.72	5.69	0.08	0.436	0.378	0.204

^a, b^—statistically significant differences at *p* ≤ 0.05.

**Table 5:** Color parameters (CIE L*a*b*) of meat (m. longissimus lumborum).

Item	Prebiotic Supplement (Pre)			Probiotic Supplement (Pro)		SEM	*p*-Value		
	Control 0	Inulin 20	DJA 40	−	+		Pre	Pro	Interaction
Fresh meat:									
L*—lightness	52.00	55.16	54.46	53.93	53.82	4.64	0.132	0.664	0.452
a*—redness	19.19 ^a^	16.21 ^b^	17.82 ^ab^	17.82	17.66	0.86	0.044	0.457	0.109
b*—yellowness	1.47 ^b^	2.02 ^a^	1.89 ^ab^	1.78	1.80	0.16	0.041	0.495	0.125
C*—chroma	19.50 ^a^	16.85 ^b^	19.77 ^a^	18.71	18.70	0.92	0.038	0.826	0.105
h°—hue angle	4.35 ^c^	5.30 ^a^	4.75 ^b^	4.77	4.83	0.24	0.044	0.458	0.128
Cooked meat:									
L*—lightness	69.49	70.05	69.30	69.89	69.33	3.46	0.254	0.441	0.352
a*—redness	11.22	10.89	11.23	11.10	11.12	0.86	0.204	0.848	0.179
b*—yellowness	3.64	3.46	3.51	3.54	3.53	0.14	0.069	0.906	0.231
C*—chroma	11.55	11.55	11.69	11.60	11.58	0.85	0.306	0.696	0.189
h°—hue angle	17.35	16.96	17.35	17.13	17.30	1.01	0.302	0.492	0.145

^a, b, c^—statistically significant differences at *p* ≤ 0.05.

**Table 6:** Texture parameters and sensory meat quality of cooked meat (m. longissimus lumborum).

Item	Prebiotic Supplement (Pre)			Probiotic Supplement (Pro)		SEM	*p*-Value		
	Control 0	Inulin 20	DJA 40	−	+		Pre	Pro	Interaction
Color	4.7	4.8	4.7	4.6	4.7	0.01	0.469	0.409	0.286
Odour	4.5	4.6	4.6	4.5	4.6	0.01	0.608	0.595	0.394
Palatability	4.4	4.6	4.6	4.4	4.6	0.01	0.166	0.345	0.175
Consistency	4.8	4.8	4.8	4.8	4.8	0.01	0.958	0.984	0.938
Shear force, N max	64.00 ^a^	53.35 ^b^	60.10 ^ab^	59.20	59.10	2.86	0.036	0.588	0.042
Shear energy, J	0.26 ^a^	0.19 ^b^	0.20 ^b^	0.21	0.22	0.01	0.039	0.356	0.212
Hardness, N	121.45 ^a^	74.20 ^b^	77.85 ^b^	92.13	90.20	3.98	0.004	0.159	0.112
Springiness, mm	0.57	0.55	0.55	0.55	0.56	0.02	0.378	0.448	0.254
Gumminess, N	38.15 ^a^	25.45 ^c^	30.50 ^b^	31.70	31.03	1.01	0.006	0.372	0.162
Chewiness, N × mm	21.50 ^a^	13.85 ^c^	16.65 ^b^	17.43	17.23	0.82	0.004	0.459	0.242

^a, b, c^—statistically significant differences at *p* ≤ 0.05.

**Table 7:** Parameters of water-holding capacity and texture of meat (m. longissimus lumborum).

Item	Prebiotic Supplement (Pre)			Probiotic Supplement (Pro)		SEM	*p*-Value		
	Control 0	Inulin 20	DJA 40	−	+		Pre	Pro	Interaction
Drip loss, %	3.50	3.48	3.46	3.49	3.46	0.12	0.419	0.597	0.346
Cooking loss, %	28.19	27.07	27.22	27.54	27.44	1.32	0.108	0.766	0.154
Free water, mg	73.11 ^a^	64.07 ^b^	61.79 ^b^	66.53	66.11	2.26	0.039	0.449	0.244
M/T × 100 *	24.70 ^b^	27.51 ^ab^	29.23 ^a^	26.65	27.64	1.54	0.041	0.095	0.123

^a, b^—statistically significant differences at *p* ≤ 0.05; * M/T—proportion = meat surface/total area × 100.

**Table 8:** Fatty acid composition (g per 100 g of all estimated fatty acids) in meat (m. longissimus lumborum).

Item	Prebiotic Supplement (Pre)			Probiotic Supplement (Pro)		SEM	*p*-Value		
	Control 0	Inulin 20	DJA 40	−	+		Pre	Pro	Interaction
C 14:0	1.51	1.58	1.55	1.55	1.53	0.21	0.296	0.493	0.225
C 16:0	26.34	26.27	26.28	26.36	26.23	0.73	0.659	0.691	0.324
C 16:1 n-7	4.23 ^ab^	4.08 ^b^	4.38 ^a^	4.21	4.25	0.21	0.041	0.438	0.139
C 18:0	11.99	11.68	11.64	11.87	11.66	0.62	0.438	0.347	0.184
C 18:1 n-9	43.91	44.18	44.26	43.80	44.44	1.85	0.196	0.134	0.251
C 18:2 n-6	8.65	8.84	8.75	9.07 ^a^	8.42 ^b^	0.53	0.142	0.047	0.073
C 18:3 n-3	0.28	0.29	0.30	0.29	0.29	0.09	0.489	0.894	0.318
C 20:0	0.16	0.16	0.14	0.16	0.14	0.03	0.342	0.373	0.251
C 20:1 n-11	0.64	0.66	0.66	0.63	0.67	0.13	0.574	0.393	0.361
C 20:2 n-6	0.19	0.22	0.20	0.19	0.22	0.08	0.137	0.149	0.246
C 20:4 n-6	0.59	0.57	0.55	0.56	0.57	0.12	0.249	0.688	0.106
C 22:2 n-6	0.31	0.37	0.38	0.36	0.34	0.09	0.064	0.091	0.267
SFA	40.06	39.77	39.69	40.02	39.66	0.52	0.316	0.369	0.368
MUFA	48.77	48.92	49.30	48.64	49.35	0.79	0.108	0.103	0.158
PUFA	10.02	10.28	10.18	10.47 ^a^	9.84 ^b^	0.28	0.248	0.043	0.274
PUFA n-3	0.28	0.29	0.30	0.29	0.29	0.09	0.489	0.904	0.318
PUFA n-6	9.73	9.99	9.87	10.18 ^a^	9.54 ^b^	0.55	0.143	0.044	0.273
PUFA n-6/n-3	34.56 ^a^	34.84 ^a^	33.34 ^b^	35.38 ^a^	33.11 ^b^	0.43	0.047	0.039	0.082
AI	0.56	0.56	0.55	0.55	0.55	0.01	0.275	0.942	0.824
TI	1.33	1.30	1.29	1.31	1.30	0.02	0.125	0.286	0.127
h/H	1.92	1.94	1.94	1.93	1.94	0.03	0.339	0.511	0.208

^a, b^—statistically significant differences at *p* ≤ 0.05; SFA—sum of saturated fatty acids; MUFA—sum of monounsaturated fatty acids; PUFA—sum of polyunsaturated fatty acids; AI—atherogenicity index; TI—thrombogenicity index; h/H—hypocholesterolemic/hypercholesterolemic ratio.

**Table 9:** Fatty acid composition (g per 100 g of all estimated fatty acids) in backfat.

Item	Prebiotic Supplement (Pre)			Probiotic Supplement (Pro)		SEM	*p*-Value		
	Control 0	Inulin 20	DJA 40	−	+		Pre	Pro	Interaction
C 14:0	1.46	1.38	1.33	1.40	1.38	0.05	0.092	0.309	0.202
C 16:0	26.04	25.74	25.56	25.83	25.72	0.68	0.452	0.598	0.128
C 16:1 n-7	2.12	2.14	2.18	2.13	2.16	0.04	0.354	0.432	0.312
C 18:0	16.71	16.47	16.16	16.44	16.45	0.73	0.138	0.847	0.041
C 18:1 n-9	38.29	38.52	39.06	38.63	38.61	0.84	0.097	0.841	0.251
C 18:2 n-6	12.46	12.62	12.68	12.59	12.57	0.14	0.248	0.895	0.372
C 18:3 n-3	0.79	0.81	0.81	0.79	0.82	0.02	0.324	0.331	0.712
C 20:0	0.25	0.28	0.26	0.26	0.26	0.01	0.248	0.951	0.374
C 20:1 n-11	0.58	0.58	0.60	0.59	0.58	0.03	0.572	0.792	0.576
C 20:2 n-6	0.10	0.13	0.12	0.11	0.11	0.01	0.474	0.942	0.344
C 20:4 n-6	0.19	0.20	0.18	0.18	0.19	0.01	0.304	0.426	0.416
C 22:2 n-6	0.12	0.14	0.13	0.13	0.13	0.01	0.468	0.925	0.468
SFA	44.55 ^a^	43.94 ^ab^	43.37 ^b^	44.02	43.88	0.52	0.046	0.149	0.039
MUFA	40.99	41.24	41.84	41.35	41.36	0.59	0.072	0.839	0.158
PUFA	13.64	13.88	13.91	13.80	13.82	0.13	0.317	0.821	0.572
PUFA n-3	0.79	0.81	0.81	0.79	0.82	0.02	0.108	0.109	0.212
PUFA n-6	12.85	13.07	13.10	13.01	13.00	0.12	0.284	0.845	0.434
PUFA n-6/n-3	16.27	16.14	16.24	16.50	15.93	0.44	0.244	0.086	0.482
AI	0.59	0.57	0.56	0.57	0.57	0.02	0.148	0.845	0.372
TI	1.51 ^a^	1.47 ^ab^	1.44 ^b^	1.48	1.47	0.02	0.039	0.427	0.412
h/H	1.88 ^b^	1.92 ^ab^	1.96 ^a^	1.92	1.92	0.007	0.038	0.934	0.094

^a, b^—statistically significant differences at *p* ≤ 0.05; SFA—sum of saturated fatty acids; MUFA—sum of monounsaturated fatty acids; PUFA—sum of polyunsaturated fatty acids; AI—atherogenicity index; TI—thrombogenicity index; h/H—hypocholesterolemic/hypercholesterolemic ratio.

## Data Availability

The data supporting reported results are in the possession of the authors (E.R.G., M.F., G.S., M.B., and M.S).

## References

[1] Samanta A.K., Jayapal N., Senani S., Kolte A.P., Sridhar M. (2013). Prebiotic Inulin: Useful Dietary Adjuncts to Manipulate the Livestock Gut Microflora. Braz. J. Microbiol..

[2] Niness K.R. (1999). Inulin and Oligofructose: What Are They?. J. Nutr..

[3] Verdonk J.M.A.J., Shim S.B., van Leeuwen P., Verstegen M.W.A. (2005). Application of Inulin-Type Fructans in Animal Feed and Pet Food. Br. J. Nutr..

[4] Rossi M., Corradini C., Amaretti A., Nicolini M., Pompei A., Zanoni S., Matteuzzi D. (2005). Fermentation of Fructooligosaccharides and Inulin by Bifidobacteria: A Comparative Study of Pure and Fecal Cultures. Appl. Environ. Microbiol..

[5] Gibson G.R., Roberfroid M.B. (1995). Dietary Modulation of the Human Colonic Microbiota: Introducing the Concept of Prebiotics. J. Nutr..

[6] Bogusławska-Tryk M., Piotrowska A., Burlikowska K. (2012). Dietary fructans and their potential beneficial influence on health andperformance parametrs in broiler chickens. J. Centr. Eur. Agric..

[7] Crittenden R.G., Playne M.J. (1996). Production, Properties and Applications of Food-Grade Oligosaccharides. Trends Food Sci. Technol..

[8] Dibner J.J., Richards J.D. (2005). Antibiotic Growth Promoters in Agriculture: History and Mode of Action. Poult. Sci..

[9] Dhama K., Mahendran M., Tomar S., Chauhan R. (2008). Beneficial Effects of Probiotics and Prebiotics in Livestock and Poultry: The Current Perspectives. Polivet.

[10] Macfarlane G.T., Cummings J.H. (1999). Probiotics and Prebiotics: Can Regulating the Activities of Intestinal Bacteria Benefit Health?. BMJ.

[11] Gaafar A.M., Serag El-Din M.F., Boudy E.A., El-Gazar H.H. (2010). Extraction Conditions of Inulin from Jerusalem Artichoke Tubers and its Effects on Blood Glucose and Lipid Profile in Diabetic Rats. J. Am. Sci..

[12] Brkljača J., Bodroža-Solarov M., Krulj J., Terzić S., Mikić A., Jeromela A.M. (2014). Quantification of Inulin Content in Selected Accessions of Jerusalem Artichoke (*Helianthus tuberosus* L.). Helia.

[13] Kirchgessner M., Roth F.X. (1983). Schätzgleichungen zur Ermittlung des energetischen Futterwertes von Mischfuttermitteln für Schweine. J. Anim. Physiol. Anim. Nutr..

[14] NRC (2021). Nutrient Requirements of Swine.

[15] EU (2005). Council Regulation (EC) No 1/2005 of 22 December 2004 on the protection of animals during transport and related operations and amending Directives 64/432/EEC and 93/119/EC and Regulation (EC) No 1255/97. Off. J. Eur. Union.

[16] EU (2009). Council Regulation (EC) No 1099/2009 of 24 September 2009 on the protection of animals at the time of killing. Off. J. Eur. Union.

[17] Różycki M., Tyra M. (2010). The procedure of pigs fattening and slaughter value estimation at Swine Performance Testing Stations. Report of pig breeding in Poland. Report of Pig Breedingin Poland.

[18] Horwitz W., Latimer G.W. (2011). Official Methods of Analysis of AOAC International.

[19] Davies M.G., Thomas A.J. (1973). An investigation of hydrolytic techniques for the amino acid analysis of foodstuffs. J. Sci. Food Agric..

[20] Schram E., Moore S., Bigwood E.J. (1954). Chromatographic Determination of Cystine as Cysteic Acid. Biochem. J..

[21] Grela E.R., Świątkiewicz M., Kowalczuk-Vasilev E., Florek M., Kosior-Korzecka U., Skałecki P. (2020). An Attempt of Implementation of Immunocastration in Swine Production—Impact on Meat Physicochemical Quality and Boar Taint Compound Concentration in the Meat of Two Native Pig Breeds. Livest. Sci..

[22] CIE (2004). Colorimetry.

[23] Witte V.C., Krause G.F., Bailey M.E. (1970). A new extraction method for determining 2-thiobarbituric acid values of pork and beef during storage. J. Food Sci..

[24] Grau R., Hamm R. (1953). Eine einfache Methode zur Bestimmung der Wasserbindung im Muskel. Naturwissenschaften.

[25] Hofmann K., Hamm R., Blüuchel E. (1982). Neues uber die Bestimmung der Wasserbindung des Fleisches mit Hilfe der Filterpapierpreßmethode. Fleischwirtschaft.

[26] Florek M., Junkuszew A., Bojar W., Skalecki P., Gregula-Kania M., Litwinczuk A., Gruszecki T.M. (2016). Effect of Vacuum Ageing on Instrumental and Sensory Textural Properties of Meat from Uhruska Lambs. Ann. Anim. Sci..

[27] Folch J., Lees M., Stanley G.H.S. (1957). A simple method for the isolation and purification of total lipides from animal tissues. J. Biol. Chem..

[28] PN-EN ISO 12966-2:2017-05. https://sklep.pkn.pl/pn-en-iso-12966-2-2017-05e.html.

[29] PN-EN ISO 12966-1:2015-01. https://sklep.pkn.pl/pn-en-iso-12966-1-2015-01e.html.

[30] ISO 12966-2:2017. https://www.iso.org/cms/render/live/en/sites/isoorg/contents/data/standard/07/21/72142.html.

[31] Samolińska W., Kowalczuk-Vasilev E., Grela E.R. (2018). Comparative effect of different dietary inulin sources and probiotics on growth performance and blood characteristics in growing-finishing pigs. Arch. Anim. Nutr..

[32] Ulbricht T.L.V., Southgate D.A.T. (1991). Coronary Heart Disease: Seven Dietary Factors. Lancet.

[33] Fernández M., Ordóñez J.A., Cambero I., Santos C., Pin C., Hoz L. (2007). de la Fatty Acid Compositions of Selected Varieties of Spanish Dry Ham Related to Their Nutritional Implications. Food Chem..

[34] Matuszewska I., Baryłko-Pikielna N. (1995). The Effect of Sample Exposure Time on the Time Intensity Response to NaCl Solutions. Food Qual. Prefer..

[35] Greguła-Kania M., Gruszecki T.M., Junkuszew A., Juszczuk-Kubiak E., Florek M. (2019). Association of CAST Gene Polymorphism with Carcass Value and Meat Quality in Two Synthetic Lines of Sheep. Meat Sci..

[36] PN-ISO 4121:1998 (1998). Sensory Analysis—Methodology—Evaluation of Food Products by Methods Using Scales.

[37] StatSoft Releases STATISTICA Version 10 Analytics Solutions. http://www.qualitydigest.com/inside/six-sigma-news/statsoft-releases-statistica-version-10-analytics-solutions-012311.html.

[38] Żak G., Pieszka M. (2009). Improving Pork Quality through Genetics and Nutrition. Ann. Anim. Sci..

[39] Fang L.H., Jin Y.H., Do S.H., Hong J.S., Kim B.O., Han T.H., Kim Y.Y. (2019). Effects of Dietary Energy and Crude Protein Levels on Growth Performance, Blood Profiles, and Nutrient Digestibility in Weaning Pigs. Asian-Australas. J. Anim. Sci..

[40] Swiatkiewicz S., Swiatkiewicz M., Arczewska-Wlosek A., Jozefiak D. (2015). Chitosan and Its Oligosaccharide Derivatives (Chito-Oligosaccharides) as Feed Supplements in Poultry and Swine Nutrition. J. Anim. Physiol. Anim. Nutr..

[41] Liao S.F., Nyachoti M. (2017). Using Probiotics to Improve Swine Gut Health and Nutrient Utilization. Anim. Nutr..

[42] Markowiak P., Śliżewska K. (2018). The Role of Probiotics, Prebiotics and Synbiotics in Animal Nutrition. Gut Pathog..

[43] Chlebicz-Wójcik A., Śliżewska K. (2020). The Effect of Recently Developed Synbiotic Preparations on Dominant Fecal Microbiota and Organic Acids Concentrations in Feces of Piglets from Nursing to Fattening. Animals.

[44] Yu M., Li Z., Rong T., Wang G., Liu Z., Chen W., Li J., Li J., Ma X. (2020). Different Dietary Starch Sources Alter the Carcass Traits, Meat Quality, and the Profile of Muscle Amino Acid and Fatty Acid in Finishing Pigs. J. Anim. Sci. Biotechnol..

[45] Wang W., Chen D., Yu B., Huang Z., Luo Y., Zheng P., Mao X., Yu J., Luo J., He J. (2019). Effect of Dietary Inulin Supplementation on Growth Performance, Carcass Traits, and Meat Quality in Growing–Finishing Pigs. Animals.

[46] Wang W., Chen D., Yu B., Huang Z., Mao X., Zheng P., Luo Y., Yu J., Luo J., Yan H. (2020). Effects of Dietary Inulin Supplementation on Growth Performance, Intestinal Barrier Integrity and Microbial Populations in Weaned Pigs. Br. J. Nutr..

[47] Birmani M.W., Nawab A., Ghani M.W., Li G., Xiao M., An L. (2019). A review: Role of inulin in animal nutrition. J. Food Technol. Res..

[48] Huff-Lonergan E., Baas T.J., Malek M., Dekkers J.C.M., Prusa K., Rothschild M.F. (2002). Correlations among Selected Pork Quality Traits. J. Anim. Sci..

[49] Sieczkowska H., Kocwin-Podsiadla M., Antosik K., Krzecio E., Zybert A., Korszen L. (2010). Charakterystyka jakości surowca wieprzowego wybranych grup rasowych tuczników. Rocz. Nauk. Pol. Tow. Zootech..

[50] van Laack R.L.J.M., Kauffman R.G. (1999). Glycolytic Potential of Red, Soft, Exudative Pork Longissimus Muscle1. J. Anim. Sci..

[51] Tarladgis B.G., Watts B.M., Younathan M.Y., Dugan L. (1960). A distillation method for the quantitative determination of malonaldehyde in rancid foods. J. Am. Oil Chem. Soc..

[52] Min B., Nam K.C., Cordray J., Ahn D.U. (2008). Endogenous factors affecting oxidative stability of beef loin, pork loin, and chicken breast and thigh meats. J. Food Sci..

[53] Lindahl G., Lundström K., Tornberg E. (2001). Contribution of Pigment Content, Myoglobin Forms and Internal Reflectance to the Colour of Pork Loin and Ham from Pure Breed Pigs. Meat Sci..

[54] Herosimczyk A., Lepczyński A., Ożgo M., Barszcz M., Jaszczuk-Kubiak E., Pierzchała M., Tuśnio A., Skomiał J. (2017). Hepatic Proteome Changes Induced by Dietary Supplementation with Two Levels of Native Chicory Inulin in Young Pigs. Livest. Sci..

[55] Hansen L.L., Stolzenbach S., Jensen J.A., Henckel P., Hansen-Møller J., Syriopoulos K., Byrne D.V. (2008). Effect of Feeding Fermentable Fibre-Rich Feedstuffs on Meat Quality with Emphasis on Chemical and Sensory Boar Taint in Entire Male and Female Pigs. Meat Sci..

[56] Hansen L.L., Agerhem H., Rosenvold K., Jensen M.T. (2002). Effect of Brussels Sprouts and Inulin/Rape Seed Cake on the Sensory Profile of Pork M. Longissimus Dorsi. Meat Sci..

[57] Kjos N.P., Øverland M., Fauske A.K., Sørum H. (2010). Feeding Chicory Inulin to Entire Male Pigs during the Last Period before Slaughter Reduces Skatole in Digesta and Backfat. Livest. Sci..

[58] Rideout T.C., Fan M.Z., Cant J.P., Wagner-Riddle C., Stonehouse P. (2004). Excretion of Major Odor-Causing and Acidifying Compounds in Response to Dietary Supplementation of Chicory Inulin in Growing Pigs1. J. Anim. Sci..

[59] Byrne D.V., Thamsborg S.M., Hansen L.L. (2008). A Sensory Description of Boar Taint and the Effects of Crude and Dried Chicory Roots (Cichorium Intybus L.) and Inulin Feeding in Male and Female Pork. Meat Sci..

[60] Iwańska E., Mikołajczak B., Grześ B., Pospiech E. (2016). Impact of post mortem aging of pork on changes in the isoelectric point of the proteins and tenderness. Med. Weter..

[61] Rosenvold K., Lærke H.N., Jensen S.K., Karlsson A.H., Lundström K., Andersen H.J. (2001). Strategic Finishing Feeding as a Tool in the Control of Pork Quality. Meat Sci..

[62] Aluwé M., Langendries K.C.M., Bekaert K.M., Tuyttens F.A.M., Brabander D.L.D., Smet S.D., Millet S. (2013). Effect of Surgical Castration, Immunocastration and Chicory-Diet on the Meat Quality and Palatability of Boars. Meat Sci..

[63] Brestenský M., Nitrayová S., Patráš P., Heger J., Nitray J. (2016). Effect of the Supplementation Linseed Oil, Inulin and Horse Chestnut into a High Fat Diet on the Fatty Acid Profile of Pigs. Ciênc. Rural.

[64] Chang S.Y., Belal S.A., Kang D.R., Il Choi Y., Kim Y.H., Choe H.S., Heo J.Y., Shim K.S. (2018). Influence of Probiotics-Friendly Pig Production on Meat Quality and Physicochemical Characteristics. Korean J. Food Sci. Anim. Resour..

[65] Juárez-Silva M.E., Cuchillo-Hilario M., Villarreal-Delgado E., Juárez-Silva M.E., Cuchillo-Hilario M., Villarreal-Delgado E. (2019). Dietary Supplementation of Inulin or Flavomycin and Type of Cut of Rabbit Meat: Changes on Fatty Acid Profile and Sensorial Characteristics. Rev. Mex. Cienc. Pecu..

[66] Grela E.R., Sobolewska S., Rozinski T. (2014). Effect of Inulin Extracts or Inulin-Containing Plant Supplement on Blood Lipid Indices and Fatty Acid Profile in Fattener Tissues. Pol. J. Vet. Sci..

[67] Mattioli S., Cardinali R., Balzano M., Pacetti D., Castellini C., Dal Bosco A., Frega N.G. (2017). Influence of Dietary Supplementation with Prebiotic, Oregano Extract, and Vitamin E on Fatty Acid Profile and Oxidative Status of Rabbit Meat. J. Food Qual..

